# Supplementary figures and images for: Exploring the binding properties and structural stability of an opsin in the chytrid Spizellomyces punctatus using comparative and molecular modeling
Source: PeerJ. 2017 Apr 27;5:e3206. doi: 10.7717/peerj.3206 (PMC5410147; doi:10.7717/peerj.3206)

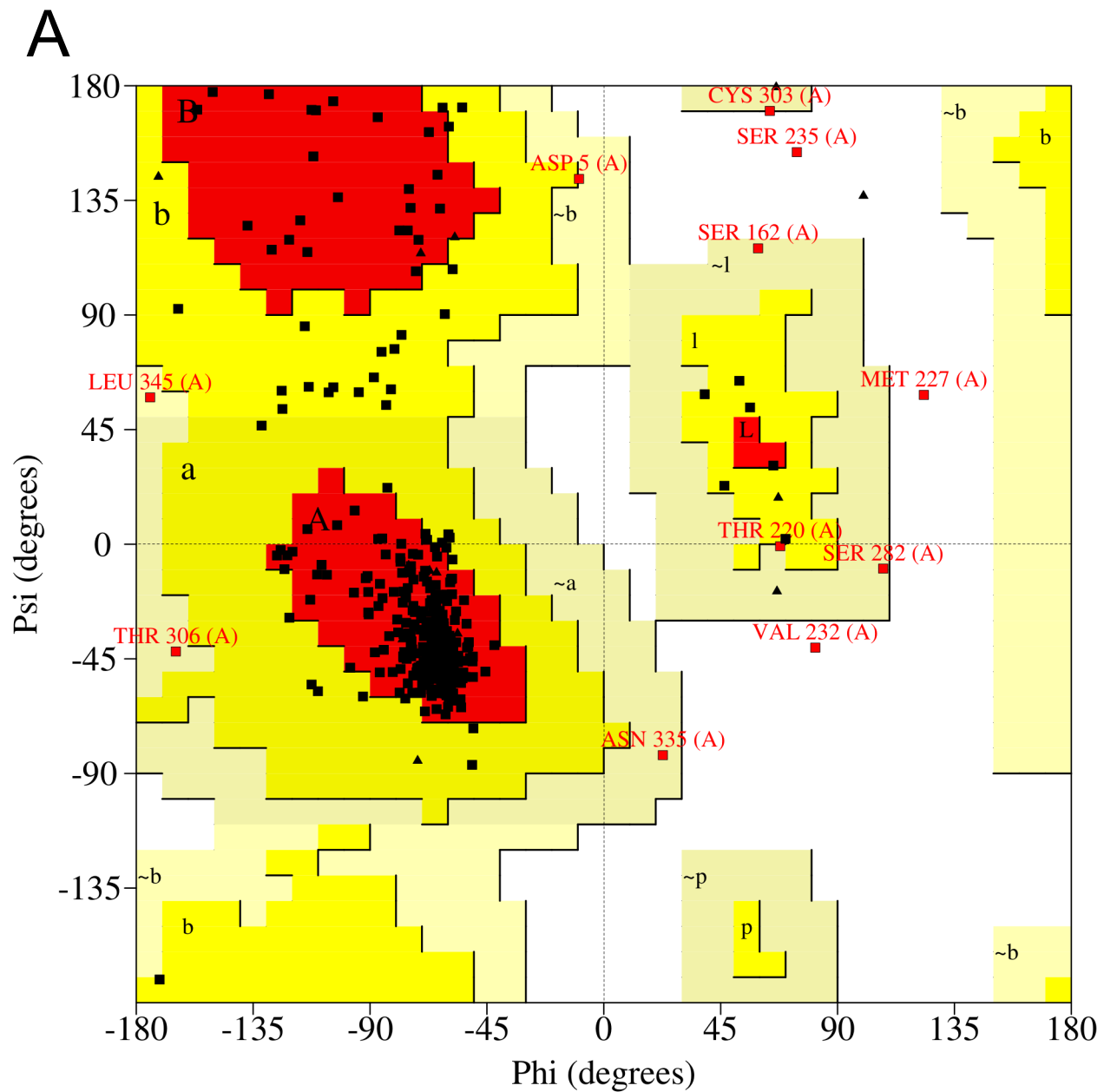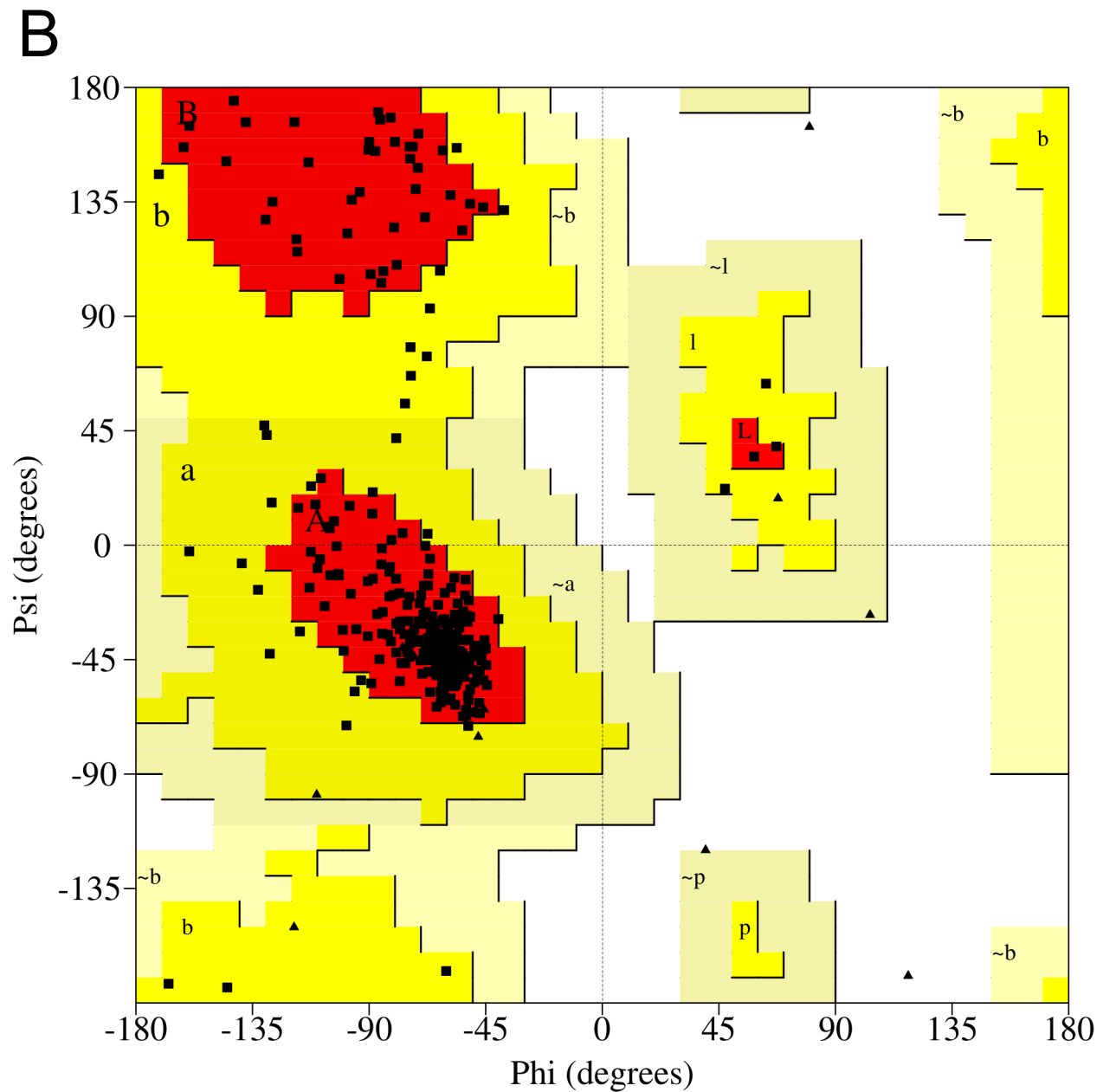

Supplement: Figure S1 — A) S. punctatus iTasser homology model and B) T. pacificus rhodopsin x-ray crystal structure (PDB ID: 2Z73). [file peerj-05-3206-s001.pdf]

**A**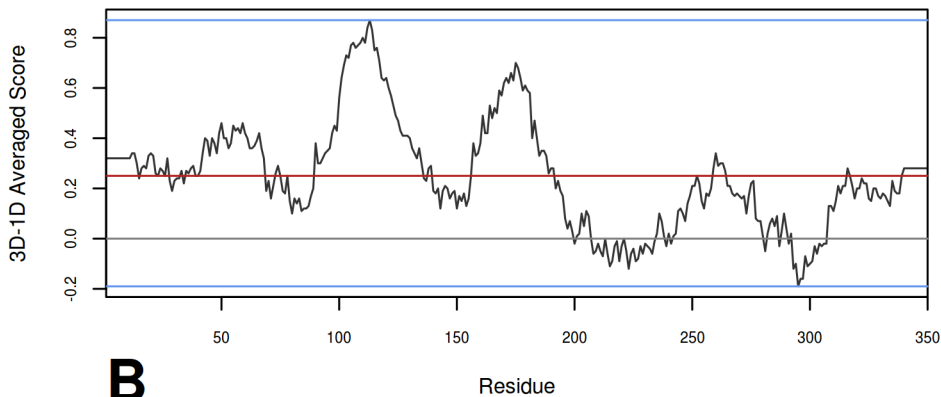**B**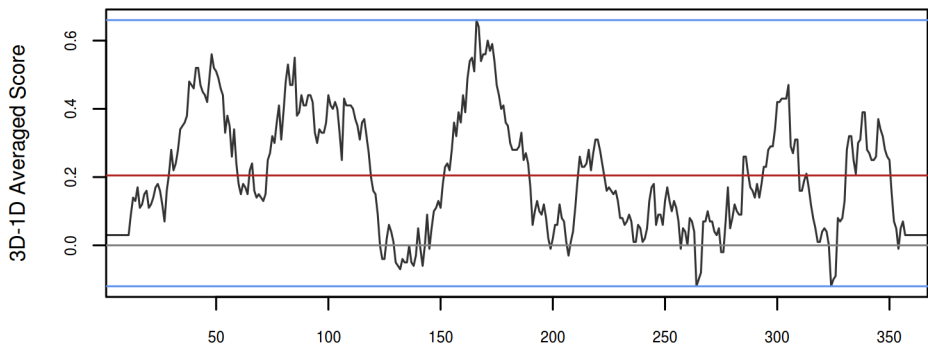**C**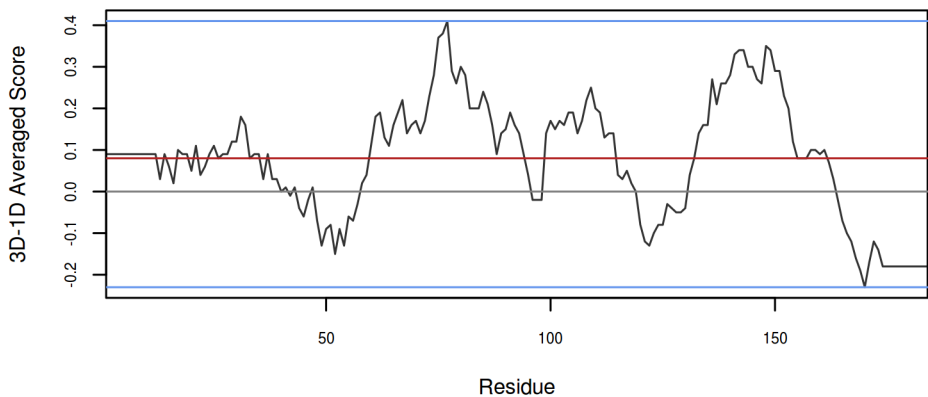

Supplement: Figure S2 — (A) the T. pacificus crystal structure, (B) the S. punctatus homology model against the iTasser GPCR database, and (C) the S. punctatus homology model against the sensory rhodopsin II xray crystal structure from the archaeon Natronomonas pharaonis (PDBid 1H68). Far more residues have averaged scores below 0 in the S. punctatus model using 1H68 as the template than in the other two structures. [file peerj-05-3206-s002.pdf]

**A**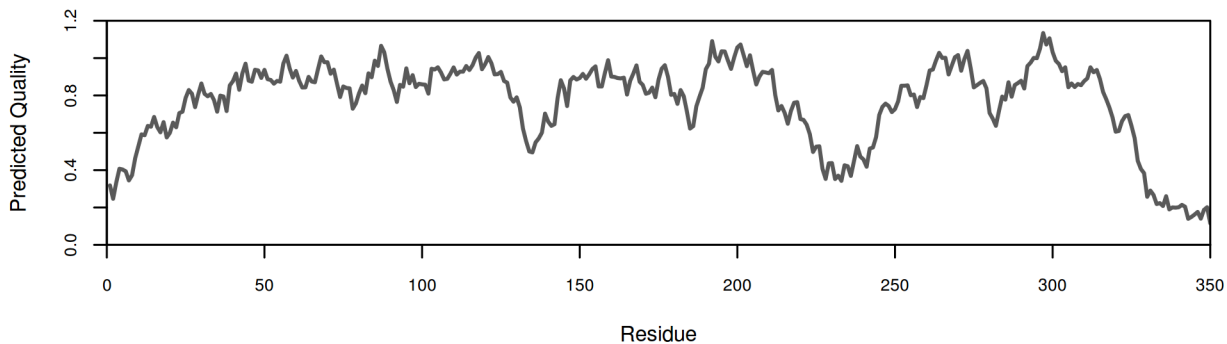**B**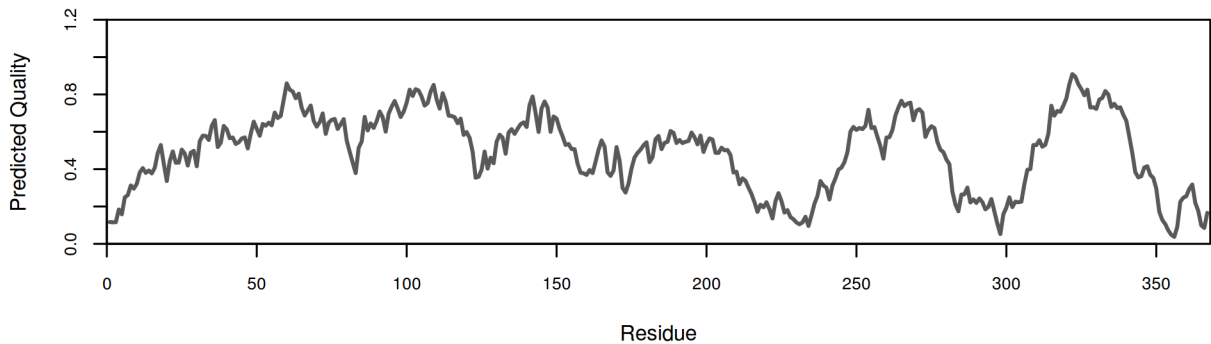**C**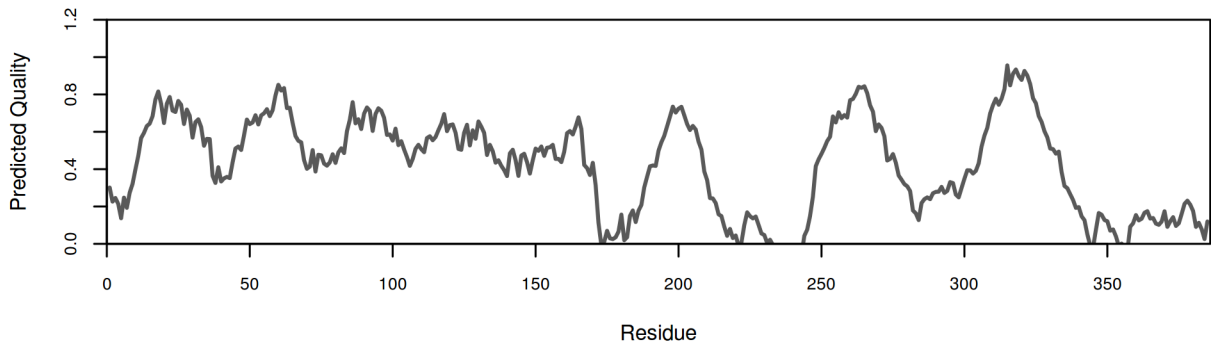

Supplement: Figure S3 — (A) the T. pacificus crystal structure, (B) the S. punctatus homology model against the iTasser GPCR database, and (C) the S. punctatus homology model against the sensory rhodopsin II xray crystal structure from the archaeon Natronomonas pharaonis (PDBid 1H68). The global quality score for the fungal sequence against the sensory rhodopsin template was lower than that of the iTasser fungal model. [file peerj-05-3206-s003.pdf]
